# Fast diagnosis of men’s fertility using Raman spectroscopy combined with chemometric methods: An experimental study

**DOI:** 10.18502/ijrm.v19i2.8470

**Published:** 2021-02-21

**Authors:** Roudabeh Sadat Moazeni Pourasil, Kambiz Gilany

**Affiliations:** ^1^Department of Analytical Chemistry, Faculty of Chemistry, Kharazmi University, Tehran, Iran.; ^2^Reproductive Biotechnology Research Center, Avicenna Research Institute, ACECER, Tehran, Iran.; ^3^Integrative Oncology Department, Breast Cancer Research Center, Motamed Cancer Institute, ACECR, Tehran, Iran.

**Keywords:** Semen analysis, Fertility, Raman spectroscopy, Metabolomics.

## Abstract

**Background:**

Idiopathic infertile men suffer from unexplained male infertility; they are infertile despite having a normal semen analysis, a normal history, and physical examination, and when female infertility factor has been ruled out.

**Objective:**

The present study aimed to develop a metabolic fingerprinting methodology using Raman spectroscopy combined with Chemometrics to detect idiopathic infertile men vs. fertile ones by seminal plasma.

**Materials and Methods:**

In this experimental study, the seminal plasma of 26 men including 13 fertile and 13 with unexplained infertility who reffered to, Avicenna Infertility Clinic, 2018, Tehran, Iran, have been investigated. The seminal metabolomic fingerprinting was evaluated using Raman spectrometer from 100 to 4250 cm-1. The principal component analysis and discriminate analysis methods were used.

**Results:**

The total of 26 samples were divided into 20 training and 6 test sets. The Principal component analysis score plot of the training set showed that the data were perfectly divided into two sides of the plot, which statistically approves the direct effect of semen metabolome changes on the Raman spectra. A classification model was constructed by linear discriminant analysis using the training set and evaluated by the test group which resulted in completely correct classification. While three of the six test samples appeared in the fertile group, the rest appeared in the infertile as expected.

**Conclusion:**

Metabolic fingerprinting of seminal plasma using Raman spectroscopy combined with chemometric classification methods accurately discriminated between the idiopathic infertile men and the fertile ones and predicted their fertility type.

## 1. Introduction

Unexplained male infertility (UMI) introduces as infertile men despite having normal semen analysis, normal history and physical examination, and when female factor infertility has been ruled out (1). A routine semen analysis remains the backbone of the evaluation of the male factor infertility (2).

Human semen characteristics are evaluated by the World Health Organization (WHO) using semen volume, sperm concentration, sperm motility, sperm morphology (3). Related to the conventional semen analysis, it is difficult to determine a threshold to distinguish the fertile from infertile based on ejaculate results (4).

Metabolites are generally considered low-molecular-weight components (≤ 2000 Da) and include metabolic intermediates, hormones, sugars, organic acids, amino acids, lipids, nucleosides, vitamins, minerals and other signaling molecules, and secondary metabolites (5, 6). Metabolic fingerprinting or metabolomics is defined as a global, high-throughput, fast analysis to provide a sample classification. Additionally, it can be used as a screening tool to differentiate samples from different origin, that is, unhealthy/healthy (7).

In metabolic fingerprinting, several techniques were used such as high-performance liquid chromatography, nuclear magnetic resonance spectroscopy, mass spectrometry, gas chromatography, optical spectroscopy, Fourier transform-infrared, and near-infrared spectroscopies (8, 9, 10).

In addition, recently, gas chromatography-mass spectrometry has demonstrated significant changes at the metabolomic level in patients' sperm cells (11).

Not being sensitive to water and the intensity of spectral features in solution is directly proportional to the concentration of the particular specie are two important fetures of Raman spectroscopy which make it more beneficial other than optical spetctorcopies (OS). Also, human semen plasma sample preparation and analysis are quite easy, safe, and fast. The Raman frequency shifts are conventionally measured in wavelength (cm-1), depending on the atomic mass or molecular bonds of specimens (12).

In the present study, Raman spectroscopy combined with multivariate pattern recognition techniques was used to analyze human seminal plasma. The principal component analysis (PCA) as a data projection and dimension reduction technique has shown that the microscopic structure of the metabolome of seminal plasma from idiopathic infertile and fertile men are totally different and could be used to construct a valid classification model. Linear discriminate analysis (LDA) was applied as a classification technique to construct a model to predict the fertility. Classification results reveal that the analysis of seminal plasma using Raman spectroscopy combined with chemometric techniques accurately predicted the type of fertility of all test samples and can be used as a complementary fast diagnostic test for men's fertility (12).

## 2. Materials and Methods

### Sample selection

13 men with a proven normal spermogram test as assessed by the WHO Manual and 13 idiopathic infertile men who were done the infertility treatment in the Avicenna Infertility Clinic (2018, Tehran, Iran) were selected. The main criteria for classification of idiopathic infertile men was low sperm motility (< 40% motile spermatozoa; > 15 million spermatozoa/ml; > 4% normal forms).

### Metabolome extraction

Purifcation of seminal plasma was done using the centrifuge (3,000 rpm, 10 min, 25°C), and after that supernatant was collected and stored at -20°C for further analysis. 500 µL of cold methanol/water (9:1) was added to 400 µL of the semen plasma to extract the polar metabolite. The mixture was kept in 4°C for 20 min and than centrifuged for 8 min at 6,000 rpm. The supernatant was used for Raman spectroscopy (13).

### Raman spectroscopy

Almega thermo nicolet dispersive was used for Raman analysis. The following parameters and specifications of Raman spectrometr were applied: the spectral range: 100-4250 cm-1; Laser: second harmonic at 532 nm of a ND: YLF laser; Resolution: 4 cm-1; and Laser power: 30 mW in order to keep the sample safe (13). Each sample was analyzed thrice by Raman spectroscopy.

### Ethical considerations

This project is a prospective study and has been approved by the ethics committee of the Reproductive Immunology Research Center, Avicenna Research Institute, ACECER, Tehran, Iran and the letter as the supplementary material is available.

### Statistical analysis

Principale component analysis (PCA) is a method to build multivariate models by transformation and visualization of complex data sets into a new perspective in which the most relevant information is made more obvious (14, 15). PCA is a dimension-reducing technique, which projects objects and variables to low-dimensional spaces to exploratory purposes (16).

In supervised pattern recognition technique or calssifcation method, objects fall into groups when we have prior knowledge of the groups to be expected. Linear discriminant analysis (LDA) is a classifaction method which is based on the Mahalanobis distance involving the determination of a linear equation-like regression that will predict which group the case belongs to (17). Data were analyzed by PCA and LDA classification models were developed using the MATLAB version 9.0.0.341360 (R2016a).

## 3. Results

**Figure 1:**
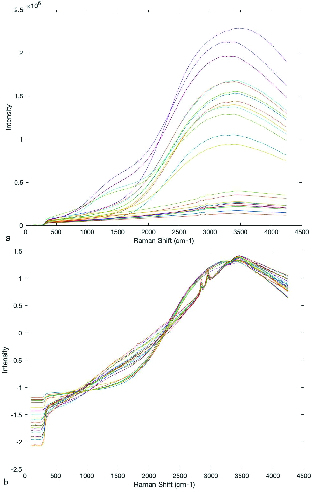
The Raman spectra (a) before and (b) after autoscaling.

In this experimental study, Raman spectroscopy combined with the multivariate pattern recognition techniques of Chemometrics was used to find the metabolome changes in the metabolome of the human semen plasma of fertile men and those with unexplained infertility. In this respect, the spectra of the metabolome of the semen plasma of a total of 26 men -13 with unexplained infertility and 13 fertile - have been investigated. The collected data include the intensity of Raman signals between 100 and 4250 cm-1, which is shown in Figure 1a. All of the Raman spectra were autoscaled prior to multivariate data analysis. Autoscaling is the most commonly used scaling method. In autoscaling, each spectrum is divided by its standard deviation as the scaling factor. Figure 1b shows the Raman spectra after autoscaling. Comparing Figure 1b with 1a demonstrates the positive effect of the autoscaling to differentiate between the Raman spectra of fertile and infertile men.

**Figure 2:**
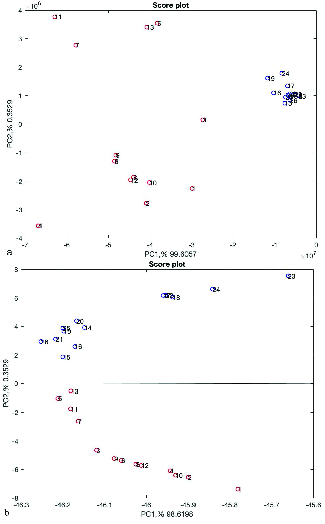
The scores plot of the training set; the red circles indicate the fertile group and the blue idiopathic infertile group (a) before and (b) after autoscaling.

The autoscaled data sets were randomly divided into two groups of training and test sets for further analysis. Accordingly, 20 samples (10 samples of fertile and 10 samples of unexplained infertile men) were included in the training set and 6 samples (3 samples of fertile and 3 samples of unexplained infertile men) in the test set. The PCA was used as a dimension reduction of data and visualization purposes. In the score plot, one PC against those of another was plotted. The most common score plot involves the scores of PC2 versus PC1. Each sample group is represented by a different colour. Figures 2a and 2b show the PCA scores plot, the PC1 versus PC2, before and after autoscaling, respectively.

The LDA method was used for supervised classification of Raman spectra data of fertile versus unexplained infertile men. The performances of classification model are defined by the classification performance (CP) equation which is as follows: 

 CP %= The  number  of  data  which  is  correctly  classified  in  the  test  set  The  whole  number  of  data  in  the  test  set ×100

In this study, the LDA classification method predicted all the test set samples correctly and the CP% for classification model was 100%.

## 4. Discussion

Combination of analytical, biochemical, and spectral analysis make it possible the identification and quantification of biomarkers which establishing the signatures of the metabolites for healthy control and illnesses gropus. Pattern recognition methods combined with OS is one of the easiest and cheapest techniques for clinical diagnosis. Here, Raman spectroscopy combined with LDA was used to model the metabolome of the human semen plasma of fertile and unexplained infertile men.

Figures 1a and 1b, respectively, show the Raman spectra of samples before and after autoscaling. Figure 1b confirms the possibility of the existence of two different types of samples. As can be seen around 2800-3000 cm-1, there are two dominant peaks in unexplained infertile men which does not exist in Raman spectra fertile group. This result is completely in accordance with the result of Liu *et al.* (12). The peak ratios between 2955 and 2841 cm-1 are distinguishing parts of the Raman spectra of seminal plasma from normal and abnormal semen samples. As can be seen in Figures 2a and 2b, about more than 99.5% of the total variance of data was explained with two first principal components (PC1% + PC2%). In Figure 2b, the scores of healthy and unhealthy samples were completely divided into two sides of plot which comes from autoscaling effect, however, the scores of samples in Figure 2a are spread randomly in the plot. The result of preprocessed PCA score plots, Figure 2b, statistically approve that the data of Raman spectroscopy combined with chemometrics can be applied in the metabolomics studies.

**Figure 3:**
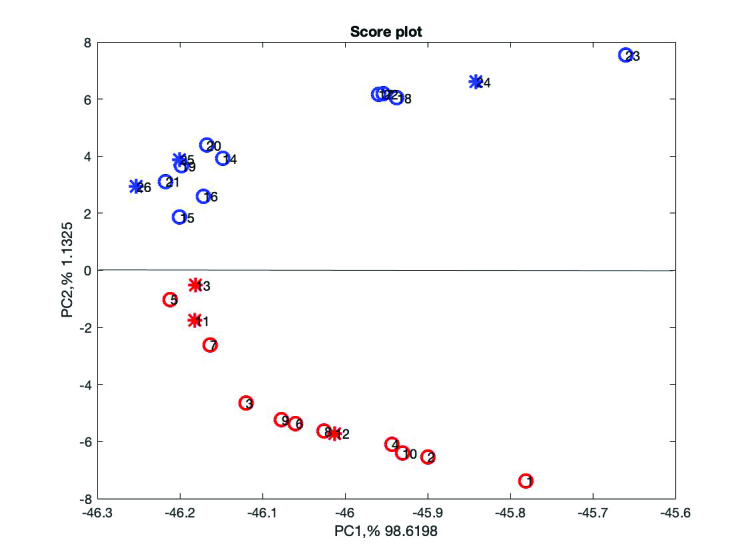
The scores plot of both sets, the training and test; the red stars indicate the fertile group and the blue idiopathic infertile group. The stars are the test set samples.

DA from Statistic Toolbox of MATLAB was implemented on the data set as the method of classification. The method with the “linear” function, LDA, was used to model the training data set. In the training set, fertile and infertile spectra were coded by 1 and 0, respectively. Next, the constructed model was used to predict the fertility type of the test set. A variable selection method was employed to get better model prediction, to improve model statistics, and to achieve more interpretable model (18). The orthogonal projection approach (OPA) was implemented for identifying selective wavelengths in Raman spectra which are responsible for fertility and idiopathic infertility. The extensive description of OPA theory can be found in the literature (19). Figure 3 shows the PCA scores plot, the PC1 versus PC2, including the test set samples. From the six test samples, three appeared in the fertile group and three in the idiopathic infertile group. The test samples are presented in circles.

As can be shown in Figure 3, the test set samples appeared correctly in its group, samples 11-13 were the fertile test set and appeared in the lower part of Figure 3 in the fertile group, and accordingly samples 24-26 were the infertile test set and appeared in the upper part of Figure 3 in the infertile group. In Raman spectroscopy the sample preparation is not necessary, it does not destroy specimens, and is less time-consuming (20, 21).

## 5. Conclusion

PCA score plot of the preprocessed data after autoscaling statistically approved the direct effect of metabolome changes on the Raman spectra. LDA as a classification method was used to classify the Raman spectra of fertile and infertile men. The model was implemented on the test samples to validate the model. The result of classification model shows that the group type prediction of all test samples was determined correctly. Accordingly, the metabolic fingerprinting of semen using chemometric classification methods combined with Raman spectroscopy paves the way for developing a simple, safe, fast, cheap, and non-invasive technique to discriminate idiopathic infertile men versus fertile.

##  Conflict of Interest

The authors declare that they have not no conflict of interest.
